# Inequalities in infant mortality in Italy

**DOI:** 10.1186/s13052-018-0594-6

**Published:** 2019-01-11

**Authors:** Silvia Simeoni, Luisa Frova, Mario De Curtis

**Affiliations:** 1 Dipartimento per la produzione statistica, ISTAT, Rome, Italy; 2 Dipartimento di Pediatria, Università di Roma La Sapienza, Rome, Italy

**Keywords:** Infant mortality, Neonatal mortality, Immigrants, Perinatal care

## Abstract

**Background:**

All the children of the world should be born equal, but this is not so: even in Italy, there are striking differences already at birth. Neonatal and infant mortality are accurate indexes to assess the demographic wellbeing and quality of life of a population. The aim of the present study is to analyze the infant (IMR) and neonatal (NMR) mortality rates of Italian and foreign children and to evaluate if there is a disparity among geographical macro-areas.

**Methods:**

Data from 2006 to 2015 were collected by the Italian Statistics Bureau (ISTAT) and extracted from two different national databases, which considered i) underlying cause of death and ii) birth registry. Mortality rates were calculated using conventional definitions. The main analyses were made comparing Italian versus foreigners as a single category as well as by country origin and contrasting Northern residents versus Southern ones. Comparisons between groups were done using relative risks.

**Results:**

Data show disparity in neonatal and infant mortality among immigrant and Italian residents. In 2015, neonatal (3.0 vs. 1.8/1000) and infant (4.5 vs 2.6/1000) mortality rates were higher among foreign children compared to Italian children. Among babies born to immigrant women, there is a higher infant mortality among children born to women coming from Central and South Africa (8.2 /1000).

Inequalities are reported even among Italian regions: in Southern Italy, infant mortality is 1.4 fold higher than in Northern Italy.

**Conclusion:**

Inequalities in neonatal and infant mortality are evident between Italians and immigrants and among geographical macro-areas There is therefore urgent need for a political and social plan focusing on infancy.

**Electronic supplementary material:**

The online version of this article (10.1186/s13052-018-0594-6) contains supplementary material, which is available to authorized users.

## Background

All the children of the world should be born equal, but this is not so: even in Italy, there are striking differences already at birth.

One of the most accurate indexes to assess the demographic wellbeing and quality of life of a population is neonatal mortality (defined as the number of deaths occurring in the first 28 days of life for every 1000 live births) and infant mortality (number of deaths occurring in the first year of life for every 1000 live births) [1, 2]. In the last few years, there has been a significant decrease in infant mortality in Italy, even beyond the rates recorded in the most developed Western countries [3], although neonatal and infant mortality has not decreased homogeneously.

The most recent data from the Italian Statistics Bureau (ISTAT) show disparity in neonatal and infant mortality among immigrant and Italian residents. In 2015, neonatal (3.0 vs. 1.8/1000) and infant (4.5 vs 2.6/1000) mortality rates were higher among foreign children compared to Italian children. Among babies born to immigrant women, there is a higher infant mortality among children born to women coming from Central and South Africa (8.2 /1000).

Inequalities are reported even among Italian regions: in Southern Italy, infant mortality is 1.4 fold higher than in Northern Italy.

There are several reasons for this disparity: in addition to the well-known differences in social and economic conditions, the inadequate organization of perinatal care plays a decisive role.

The aim of the study is to investigate the inequalities in infant and neonatal mortality among ethnic and geographical groups in Italy, and to explore the specific causes of death. There is therefore urgent need for a political and social plan focusing on infancy.

Infant and neonatal mortality rates (IMR and NMR) are considered central indicators of both child and overall population health status and of the civic development of a nation [1]. In addition to their use as markers of the Quality of Care received by mothers and their babies, they are correlated to general living conditions, social well-being, and economic development. Italy has one of the lowest rates for infant mortality in the world: 3 deaths for every 1000 live births [3]. This has largely been attributed to political and health reforms, environmental and socio-economic improvement, the development of a culture of children’s rights, scientific and medical progress, and the control of previously endemic communicable diseases such as malaria, tuberculosis or measles [4].

Over the last decade, the number of immigrants has increased in Italy. In 2006, foreign residents comprised 4.5% of the total resident population, compared to 8.3% in 2015 [5]. Despite a rapid increase in immigration from low-income countries, studies on immigrants’ mortality, especially neonatal and infant, are scarce.

Live births among foreigners are 15% of all live births, but mortality in the first year of life represents 23% of deaths [4]. This phenomenon could be an indication of a variety of differences between groups, including social deprivation, access to health care, morbidity occurring as a direct result of the migration process, or factors related to culture [6, 7].

Diversely, the “Southern Question”, the macro-regional differences in economy, trade, infrastructure, health care, housing and poverty is well known phenomenon and it creates a great divide within the nation [8].

As Italy’s public health service aims at social equality in medical services, systematic disparities between ethnic population subgroups or between macro-regions are considered unacceptable.

This study aimed to investigate the inequalities in infant and neonatal mortality among ethnic and geographical groups in Italy, and to explore the specific causes of death. This is the first study on the differences between Italian and immigrate residents’ infant mortality rate.

## Methods

Data from 2006 to 2015 were collected by the Italian Statistics Bureau (ISTAT) and extracted from two different national databases, which considered i) underlying cause of death and ii) birth registry.

Mortality rates were calculated using conventional definitions: for every 1000 live births, neonatal mortality rate (NMR) as the number of deaths occurring before 28 days of age; post-neonatal mortality rate as the number of deaths occurring between 28 days to 1 year; and infant mortality rate (IMR) as the number of deaths occurring in the first year of life.

Specific causes of infant death, and neonatal and post-neonatal mortality rates were calculated dividing the number of specific-cause deaths by the number of live births during the year.

The causes of death were classified according to the ICD-10 (International Statistical Classification of Diseases and Related Health Problems).

Geographical studies were conducted considering three (North, Centre and South Italy) and five geographical areas (North-West, North-East, Center, South and Islands).

Rate ratios (RR) and 95% confidence intervals (CI) [9] were calculated to estimate the significant differences between immigrants and Italians (Immigrant IMR/ Italian citizens IMR) and geographical areas (South IMR/North IMR). RR and 95% CI were computed even for specific causes of IMR.

Children were considered Italian citizens if at least one of their parents had Italian citizenship, or immigrant residents living in Italy if neither parent had Italian citizenship.

Approximately, 10% of all deaths were of unknown nationality. They were proportionally distributed between the two groups of Italian citizens and foreigners.

For sub-group analysis, migrant background was determined according to the mother’s region of origin, grouped into six areas: 1) Northern Africa, 2) Asia, 3) European Union (UE) – EU 27, 4) extra EU 27, any other European non-EU country, 5) Other Africa and 6) America.

## Results

**Fig. 1:**
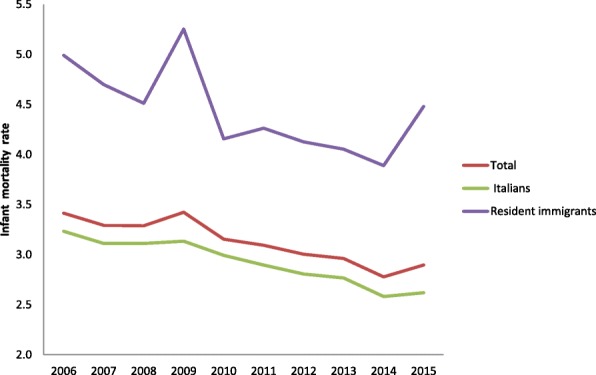
Infant mortality rate (IMR) among Italian and immigrant residents. 2006–2015

In 2015, as many as 485,780 children were born in Italy, 85% to native mothers and 15% to foreign mothers. In the same period, 1407 children died before reaching 1 year of age; of these, 77% were Italian, 23% immigrants. From 2006 to 2015, the infant mortality rate among resident immigrants was always higher than among Italians: it was 5.0 in 2006 and decreased to 4.5 in 2015, with a stable gap compared to Italian IMR (Table 1). In fact, immigrant babies had a 1.5 fold greater chance of dying than Italians, and the gap increased further in 2015 (Fig. 1).

**Table 1:** Infant mortality rates (IMR)

Year	Deaths				IMR (95%CI)				RR
	Total	Residents in Italy	Italian residents	Resident immigrants	Total	Residents	Italian residents	Immigrant residents	Immigrants/Italians
2006	2031	1912	1624	288	3.7 (3.5; 3.8)	3.4 (3.3; 3.6)	3.2 (3.1; 3.4)	5.0 (4.4; 5.6)	1.5
2007	1959	1857	1556	301	3.5 (3.3; 3.6)	3.3 (3.1; 3.4)	3.1 (3.0; 3.3)	4.7 (4.2; 5.3)	1.5
2008	1997	1896	1569	327	3.5 (3.4; 3.7)	3.3 (3.1; 3.4)	3.1 (3.0; 3.3)	4.5 (4.0; 5.0)	1.4
2009	2046	1947	1542	405	3.6 (3.5; 3.8)	3.4 (3.3; 3.6)	3.1 (3.0; 3.3)	5.3 (4.8; 5.8)	1.7
2010	1863	1773	1448	325	3.3 (3.2; 3.5)	3.2 (3.0; 3.3)	3.0 (2.8; 3.2)	4.2 (3.7; 4.6)	1.4
2011	1774	1691	1354	337	3.3 (3.1; 3.4)	3.1 (2.9; 3.2)	2.9 (2.7; 3.1)	4.3 (3.8; 4.7)	1.5
2012	1710	1605	1275	330	3.2 (3.1; 3.4)	3.0 (2.9; 3.2)	2.8 (2.7; 3.0)	4.1 (3.7; 4.6)	1.5
2013	1598	1523	1208	315	3.2 (3.0; 3.3)	3.0 (2.8; 3.1)	2.8 (2.6; 2.9)	4.1 (3.6; 4.5)	1.5
2014	1506	1396	1104	292	3.1 (2.9; 3.2)	2.8 (2.6; 2.9)	2.6 (2.4; 2.7)	3.9 (3.5; 4.4)	1.5
2015	1482	1407	1084	323	3.1 (3.0; 3.3)	2.9 (2.7; 3.1)	2.6 (2.5; 2.8)	4.5 (4.0; 5.0)	1.7

**Fig. 2:**
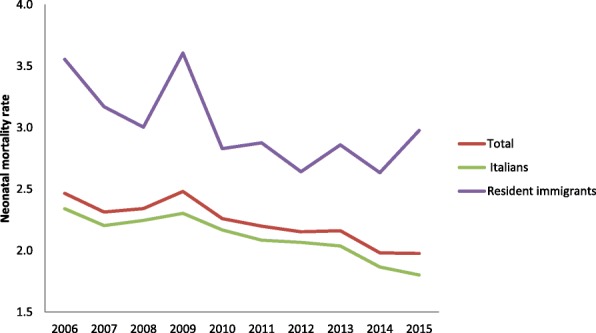
Neonatal Mortality Rate (NMR) among Italian and immigrant residents. 2006–2015

Neonatal mortality trends are similar to IMRs: immigrants have always higher rates than Italians. In the period 2010–2012 years, the gap between the two ethnic groups decreased, only to rise again afterwards (Table 2, Fig. 2). The disparity in the post-neonatal period (Post-neonatal mortality rate, PMR) was even more significant: in 2015, PMR was 0.81 for Italians and 1.5 for Immigrants (Additional file 1: Table S1).

**Table 2:** Neonatal mortality rates (NMR)

Year	Deaths				NMR(95%CI)				RR
	Total	Residents in Italy	Italian residents	Resident immigrants	Total	Residents	Italian residents	Immigrant residents	Immigrants/Italians
2006	1461	1381	1185	205	2.6(2.5;2.8)	2.5(2.3;2.6)	2.4(2.2;2.5)	3.6(3.1;4.1)	1.5
2007	1373	1305	1113	203	2.4(2.3;2.6)	2.3(2.2;2.4)	2.2(2.1;2.4)	3.2(2.7;3.6)	1.4
2008	1409	1350	1150	218	2.5(2.3;2.6)	2.3(2.2;2.5)	2.3(2.2;2.4)	3.0(2.6;3.4)	1.3
2009	1470	1411	1140	278	2.6(2.5;2.7)	2.5(2.4;2.6)	2.3(2.2;2.5)	3.6(3.2;4.1)	1.6
2010	1327	1270	1062	221	2.4(2.3;2.5)	2.3(2.1;2.4)	2.2(2.1;2.3)	2.8(2.5;3.2)	1.3
2011	1254	1202	984	227	2.3(2.2;2.5)	2.2(2.1;2.3)	2.1(2.0;2.2)	2.9(2.5;3.3)	1.4
2012	1211	1150	944	211	2.3(2.2;2.4)	2.2(2.0;2.3)	2.1(1.9;2.2)	2.6(2.3;3.0)	1.3
2013	1154	1112	899	222	2.3(2.2;2.4)	2.2(2.0;2.3)	2.1(1.9;2.2)	2.9(2.5;3.3)	1.4
2014	1055	996	803	198	2.1(2.0;2.3)	2.0(1.9;2.1)	1.9(1.8;2.0)	2.6(2.3;3.0)	1.4
2015	1001	960	757	215	2.1(2.0;2.2)	2.0(1.9;2.1)	1.8(1.7;2.0)	3.0(2.6;3.4)	1.7

**Fig. 3:**
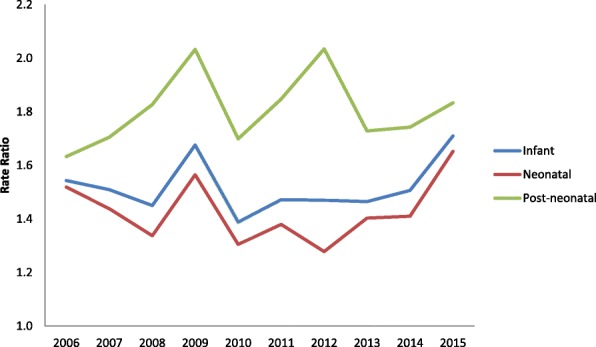
Rate ratios among Immigrants and Italians for Neonatal, Post-neonatal and Infant mortality rates

The analysis of rate ratios (RR) among immigrants and Italians for infant, neonatal and post-neonatal mortality rates showed the highest racial disparity in the post-neonatal period (Fig. 3).

Considering the last 10 years, RR trends showed an increase for all the three mortality rates in 2009 and again in 2012—but this time only for post-neonatal rate. Unfortunately, RRs have remained more or less constant, with a slight rise in 2015, showing that inequality gaps have not been filled.

**Fig. 4:**
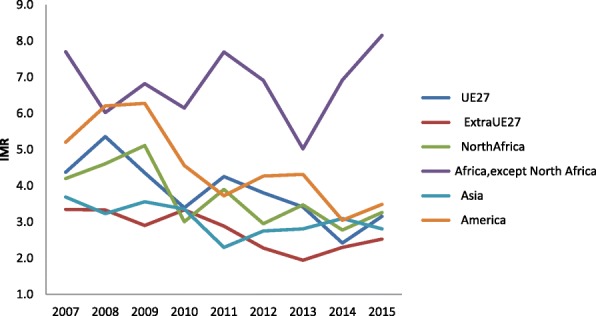
Infant mortality rate (IMR) according to mother’s nationality

Immigrant IMR varies widely according to the mother’s nationality. Babies born in Italy to women from Central and South Africa show the highest infant mortality rate – around 8 deaths per thousand live births; their IMR is similar to how the Italian situation was 25 years ago. In the last 10 years, IMR data for the different ethnic groups are improving and are converging, except for Africans, which unfortunately remains constant. Asian and extra EU babies have lower IMRs than Italians (Table 3 and Fig. 4).

**Table 3:** Infant mortality rate (IMR) according to mother’s nationality

	UE27	ExtraUE27	NorthAfrica	Africa, except North Africa	Asia	America
2006	*	*	4,2	8,4	4,2	6,1
2007	4.4	3.3	4.2	7.7	3.7	5.2
2008	5.4	3.3	4.6	6.0	3.2	6.2
2009	4.4	2.9	5.1	6.8	3.6	6.3
2010	3.4	3.3	3.0	6.1	3.4	4.6
2011	4.3	2.9	3.9	7.7	2.3	3.7
2012	3.8	2.3	3.0	6.9	2.8	4.3
2013	3.4	1.9	3.5	5.0	2.8	4.3
2014	2.4	2.3	2.8	6.9	3.1	3.0
2015	3.2	2.5	3.3	8.2	2.8	3.5

We analyzed IMRs according to the most relevant cause of death and citizenship. In the last three years, the most important causes of death were perinatal and congenital pathologies, representing 81% of all the causes of death.

During 2013–2015, immigrant babies showed mainly congenital anomalies regarding the circulatory, nervous and genital-urinary system, and the differences with Italian babies are statistically significant. Other dissimilarities are evident in diseases of the blood and immune disorders (D50-D89) and in, endocrine, nutritional and metabolic diseases (E00-E90). Among the perinatal causes, the most significant differences are in “Fetus affected by maternal factors and by complications of pregnancy, labor and delivery” (P00-P04) and “Hemorrhagic and hematological disorders of fetus and newborn” (P50-P61) diseases.

SIDS is more frequent among immigrants, with a RR of approximately 2.2 (Table 4).

**Table 4:** Specific causes of IMR: Italians vs immigrants. 2013–2015

	IMR (95%CI)		RR
	Italians	Immigrants	Immigrants/ Italians
Hemorrhagic and hematological disorders of the fetus and the newborn	0.11 (0.09; 0.13)	0.23 (0.17; 0.30)	2.1
Specific infections of the perinatal period	0.15 (0.13; 0.17)	0.19 (0.14; 0.25)	1.2
Birth asphyxia and intra-uterine hypoxia	*0.15 (0.13; 0.17)*	0.22 (0.16; 0.29)	1.4
Diseases of the circulatory system	0.08 (0.07; 0.10)	0.15 (0.10; 0.21)	1.8
Diseases of the respiratory system	0.04 (0.03; 0.06)	0.07 (0.04; 0.12)	1.7
Congenital malformations of the circulatory system	0.29 (0.26; 0.32)	0.50 (0.42; 0.61)	1.7
Congenital malformations of the nervous system	0.06 (0.04; 0.07)	0.15 (0.10; 0.21)	2.7
Fetus and new born affected by maternal factors and by complications of pregnancy, labor and delivery	0.14 (0.12; 0.16)	0.24 (0.18; 0.31)	1.7
Respiratory distress of the newborn	0.42 (0.38; 0.45)	0.53 (0.43; 0.63)	1.3
Chromosomal abnormalities, not classified elsewhere	0.06 (0.05; 0.08)	0.12 (0.08; 0.17)	1.9
Diseases of blood and hematopoietic organs and some disorders of the immune system	0.02 (0.02; 0.03)	0.06 (0.03; 0.10)	2.5
Diseases of the nervous system and sensory organs	0.07 (0.05; 0.08)	0.07 (0.04; 0.12)	1.1
Diseases of the digestive system	0.04 (0.03; 0.06)	0.06 (0.03; 0.10)	1.3
Endocrine, nutritional and metabolic diseases	0.03 (0.02; 0.04)	0.08 (0.05; 0.13)	2.8
Congenital malformations of the digestive system	0.02 (0.01; 0.03)	0.03 (0.01; 0.06)	1.7
Congenital malformations of the genitourinary system	0.03 (0.02; 0.04)	0.08 (0.05; 0.13)	2.9
Congenital malformations of the respiratory system	0.03 (0.02; 0.04)	0.05 (0.02; 0.08)	1.6
Congenital malformations and deformations of the skeletal-muscular apparatus, limbs and tegument	0.07 (0.06; 0.09)	0.11 (0.07; 0.17)	1.6
Sudden death syndrome in childhood	0.03 (0.02; 0.04)	0.07 (0.04; 0.11)	2.2

The causes of death in the post-neonatal period are different from neonatal ones. If in the first month of life perinatal diseases account for 75% of all death causes, in the post-neonatal period, they account for only 18% of all causes, with an increase in other causes of death, such as circulatory, digestive and infective diseases (Additional file 1: Table S2).

Regarding geographic distribution, in 2015 neonatal mortality in Italy was, on average, 2 per one thousand live births, with 1.8 in the north-west; 1.5 in the north-east; 2.0 in center regions; 2.3 in the south and 2.5 in the islands.

Infant mortality was on average 2.9 thousand live births, with 2.6 in the north-west; 2.4 in the north east; 2.9 in center regions; 3.3 in the south and 3.6 in the main islands (Additional file 1: Table S3).

**Fig. 5:**
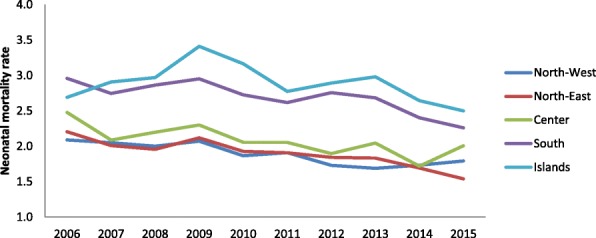
Neonatal mortality among residents in relation to geographic area

**Fig. 6:**
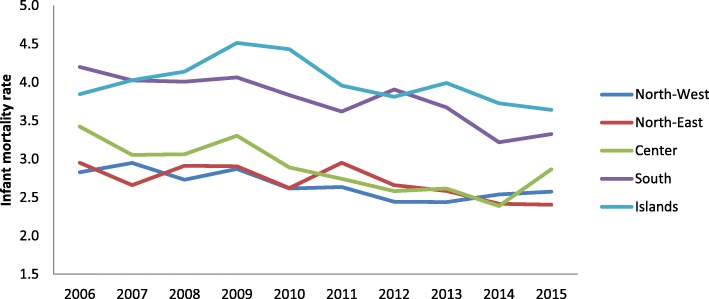
Infant mortality among residents in relation to geographic area

**Fig. 7:**
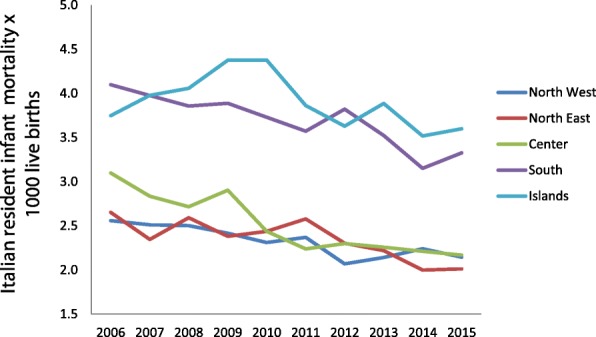
Infant mortality among Italians in relation to geographic area

**Fig. 8:**
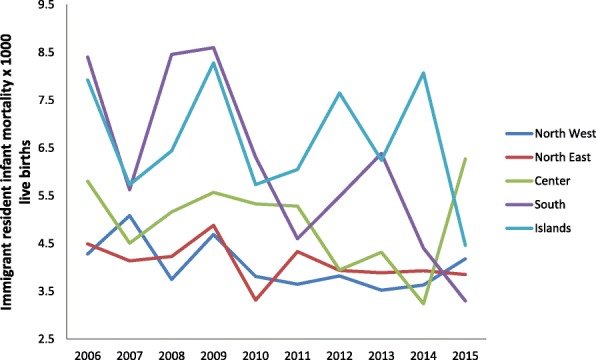
Infant mortality among Immigrants in relation to geographic area

Figures 5 and 6 show the trend for neonatal and child mortality of residents over the past 10 years. Greater mortality is evident in the southern regions and the main islands than in the central-northern regions. A child born in South Italy has around 40% of risk to dye more than a child born in North regions. The infant mortality study according to the nationality and the geographical areas (Figs. 7 and 8) shows different sceneries for Italians and immigrants: the Italian residents have a decreasing mortality in all the macro-areas even if the gap South/North persists, the immigrants have different mortality trends according to the macro-areas. Their mortality decreases slowly in the North area, faster in the South and increases in the Center.

**Fig. 9:**
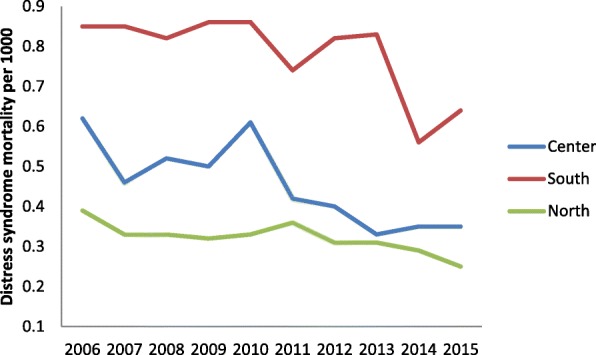
Neonatal mortality for respiratory distress syndrome and geographic area

The respiratory distress is the first cause of death during the first year of life and Fig. 9 shows the higher infant mortality rate for respiratory distress in the southern Italian regions. In all the geographical macro-areas this specific cause of death mortality is decreased, but the gap South/North has rised.

## Discussion

The data from the Italian Statistics Bureau (ISTAT) reported above, are in agreement with previous investigations highlighting a greater risk of death for babies born to immigrant women and among infants born in the south of Italy.

In 2015, the foreign children present a risk to dye of 70% more than Italian ones and children born in South Italy a risk of 40% compared to babies born in North Italy.

In both cases, the gap foreigners/Italians and South/North is not decreased is ten years.

The IMR and NMR trends are similar: they gradually decrease from 2006 to 2015, more for Italians than for foreigners and in a similar way in all the geographical areas.

However, the different values of these indicators among residents groups are worrisome.

It is difficult to explain the difference between Italian and foreign babies only using nationality, but undoubtedly citizenship carries a consistent signal over time. The high level of IMR and NMR among foreign children opens the way to different hypotheses that cannot be confirmed due to the limited explanatory power of available data.

According to the Istat data about poverty, immigrant children live in family with low social and low income status more than Italian ones. In the most open and inclusive societies, the effect of low social class on infant mortality is countered by a high level of attention to specific needs of migrant communities, which in turn brings infant mortality of migrants to the same level of that of the native population [10]. On the other hand, some ethnic groups have better IMR and NMR than native one because of “the healthy migrant effect” (i.e. the selection of very healthy women at migration). In Italy, the persistent higher mortality for foreign communities can be explained by a latent attitude of the society towards foreigners, which stresses differences and does not see a poorer health status as a problematic aspect needing intervention. The social status and family income can influence the mortality, but it is undeniable that no political, social and health plan has been developed to reduce immigrant deaths.

Effective actions to prevent inequalities at birth should be: better health care during the gestation phase for both Italians and immigrants; the implementation of accompanying courses at birth, where women (especially foreigners) can be informed about the possibilities of appropriate medical care from pregnancy to childbirth and then back home with their child; taking care of pregnant women since the first visit, with a program that includes the examinations needed to monitor the health of the mother and the fetus. The question is: is the society/policy able to take into account all the specificities of migrant populations defining appropriate strategies?

Although neonatal and child mortality in Italy has decreased, the mortality ratio between the northern and central regions on the one hand, and the southern and insular ones on the other has not changed in the last decades.

The worst prognosis in the southern and insular regions is linked not only to cultural, economic and social factors, but also to organizational problems concerning the perinatal network and the presence of many small maternity units (http://www.agenas.it/images/agenas/pne/SINTESI_PNE_2017_19_DICEMBRE.pdf).

A good organization of the medical care for the mother and newborn at birth are an integral part of the right to safe birth, and require careful supervision of the quality of birth centers also in terms of organization and management (http://www.gazzettaufficiale.it/eli/id/2011/01/18/11A00319/sg, [11]).

It is urgent and necessary to devise a program to oppose health inequalities so that people living in the south also have the right to be adequately treated. It is unacceptable that in Italy the quality of health care should depend on the region in which a person is lucky enough to be born and to live.

Minimum levels of assistance (LEAs) introduced to guarantee the fundamental principles of universality, equality and equity of the health system have not achieved the proposed end. There are clear regional differences in the provision of public prevention and health care services.

The protection of the best state of health as a fundamental right of the individual and the interest of the community should be ensured, free of charge, to everyone, including minors, (Article 32 of the Constitution and article 24, paragraph 1 of the UN Convention on the Rights of the Child and adolescent of 1989) (https://www.law.upenn.edu/journals/jil/jilp/articles/3-1_Pilnik_Lisa.pdf).

In conclusion, our study demonstrated that the challenge of providing the same level of death risk for all groups (immigrants/Italians, southern/northern citizens) is far from been reached. Inequalities in health care at birth jeopardize two fundamental human rights: the right to equality and the right to health care.

## Additional file

Additional file 1: Infant mortality additional file. (XLSX 20 kb)
